# Does Topical Tranexamic Acid Facilitate Faster Discharge Following Lung Resection? A Retrospective Cohort Analysis

**DOI:** 10.3390/jcm15093290

**Published:** 2026-04-25

**Authors:** Eylem Yentürk, Ahmet Sami Bayram

**Affiliations:** 1 Department of Thoracic Surgery, Yedikule Chest Diseases and Thoracic Surgery Education and Research Hospital, Kazlıçeşme Mahallesi Belgrat Kapı Yolu, İstanbul 34020, Türkiye; 2 Department of Thoracic Surgery, Uludağ University Faculty of Medicine, Görükle Mahallesi, Bursa 16120, Türkiye

**Keywords:** tranexamic acid, fast-track surgery, thoracic surgery, length of stay, pleural drainage, blood conservation

## Abstract

**Background/Objectives**: Managing postoperative drainage and reducing the length of hospital stays continue to represent significant challenges in thoracic surgery. While systemic antifibrinolytics are effective, concerns persist regarding neurotoxicity and thromboembolic risks. In this study, we evaluated the efficacy and safety of a unique, high-volume topical tranexamic acid (t-TXA) lavage protocol designed to optimize pleuroparenchymal contact and stabilize local hyperfibrinolysis. **Methods**: A retrospective comparative study was conducted involving 52 patients undergoing major lung resection, divided into a t-TXA group (n = 26) and a control group (n = 26). The t-TXA group received an intrathoracic lavage consisting of 5 g of tranexamic acid (TXA) diluted in 500 mL of saline, while the control group received 500 mL of saline alone. The primary outcomes included postoperative day (POD) 1 drainage volumes and length of stay (LOS). The secondary outcomes were focused on hematological parameters and safety profiles, including a structured one-year follow-up for all patients. Due to the study’s exploratory nature, primary outcomes were assessed using 95% confidence intervals for hypothesis generation rather than a priori sample size calculations. **Results**: No significant differences were observed between groups regarding sex, surgical approach, or resection type. The t-TXA group demonstrated a significantly shorter LOS (4.20 ± 1.23 days) compared to the control group (5.88 ± 2.23 days; *p* = 0.001). While POD 1 drainage was numerically lower in the t-TXA group (189.23 ± 235.06 mL) versus the control (284.23 ± 169.40 mL), this difference did not reach statistical significance (*p* = 0.101). However, exploratory correlation analysis revealed a moderate negative association between t-TXA application and POD 1 drainage (r = −0.412; *p* = 0.002). Postoperative platelet counts were significantly lower in the t-TXA group (*p* = 0.009). No thromboembolic events, late complications, or deaths occurred in either group during the one-year follow-up period. **Conclusions**: High-volume t-TXA lavage is a promising adjuvant associated with significantly shorter hospital stays and a trend toward reduced postoperative drainage. While our 12-month follow-up confirmed a favorable safety profile with no adverse events, these findings should be interpreted as preliminary and hypothesis-generating. The retrospective nature of this study precludes definitive recommendations, underscoring the need for well-powered prospective randomized trials to establish the long-term safety and clinical utility of t-TXA in thoracic surgery.

## 1. Introduction

Postoperative bleeding remains a significant and feared complication in non-cardiac thoracic surgery [1]. Major hemorrhage necessitates surgical re-exploration in 1% to 3.7% of cases and is associated with high rates of allogeneic blood transfusion, which can be required in 20% to 52% of patients undergoing major pulmonary resection [2,3]. Both excessive bleeding and subsequent transfusions represent independent risk factors for increased postoperative morbidity, including infection, sepsis, and acute renal failure. These issues lead in turn to prolonged hospitalization, increased healthcare costs, and higher mortality rates [4,5]. A primary driver of non-surgical bleeding in this context is the activation of the fibrinolytic system [6]. Surgical trauma, particularly the dissection of the pleura, results in the release of a high concentration of plasminogen activators from the serous membranes [7]. This localized hyperfibrinolysis leads to the premature breakdown of hemostatic clots and persistent oozing from the large raw surfaces created during pulmonary resection or decortication [8].

While topical tranexamic acid (t-TXA) is often presumed to be a staple in the thoracic surgeon’s armamentarium, there is a significant discrepancy between its perceived utility and actual empirical evidence of its use in the contemporary literature. Tranexamic acid (TXA) is a synthetic lysine analog that acts as an effective antifibrinolytic agent [9]. By competitively and reversibly blocking the lysine-binding sites on plasminogen, TXA inhibits its activation into plasmin, thereby preventing the degradation of the fibrin clot [10,11]. The efficacy of TXA in reducing blood loss and transfusion requirements is well established across various high-bleeding-risk specialties, most notably in cardiac and orthopedic surgery. This foundational evidence has been further reinforced by landmark clinical trials, such as the Clinical Randomisation of an Antifibrinolytic in Significant Haemorrhage 2 (CRASH-2) trial for major trauma and the World Maternal Antifibrinolytic (WOMAN) trial for postpartum hemorrhage [12,13,14,15].

Despite this extensive evidence, the optimal use of TXA in non-cardiac thoracic surgery has not been clearly defined. While systemic administration is common in other fields, it has also been associated with dose-dependent adverse effects, most notably an increased risk of postoperative seizures, particularly in cardiac surgery [16]. This concern has prompted growing interest in the topical (intrathoracic) application of TXA as a potentially safer alternative. In addition to its effect on pleuroparenchymal tissues, studies suggest that t-TXA may also be effective in controlling hemorrhage from small-to-medium-sized vessels [17].

The rationale for topical administration is compelling: it delivers a high concentration of the antifibrinolytic agent directly to the site of pleural fibrinolysis while minimizing systemic absorption and associated risks [18]. However, the clinical evidence supporting this approach in thoracic surgery is sparse, with studies having yielded conflicting results [19]. Several systematic reviews and meta-analyses, including a small number of randomized controlled trials, have suggested that t-TXA significantly reduces postoperative chest tube drainage and, in some studies, transfusion requirements [6]. For example, prospective randomized controlled trials by Sabry et al. [8] and Dell’Amore et al. [20] found that t-TXA significantly reduced postoperative bleeding and the need for blood transfusions. In contrast, another randomized controlled trial reported that while t-TXA reduced postoperative bleeding, it had no significant impact on transfusion requirements [21,22]. Furthermore, the optimal dosage for intrathoracic administration remains unknown, with published studies using varying doses from 1 g to 5 g [8,20,23].

This lack of consensus, coupled with the heterogeneity in dosing and conflicting outcomes, underscores the need for further high-quality data [5]. Therefore, our aim in this study was to evaluate the efficacy and safety of t-TXA use in thoracic surgery operations. Our primary hypothesis was that high-volume topical TXA lavage would provide superior local hemostasis, leading to reduced postoperative drainage. Secondarily to this, we hypothesized that this high-dose topical application would remain localized within the pleural space, thereby achieving clinical efficacy without altering systemic coagulation parameters, ensuring a favorable safety profile for fast-track recovery.

## 2. Materials and Methods

### 2.1. Study Design and Patient Selection

This retrospective, single-center cohort study was conducted at the Department of Thoracic Surgery, Uludağ University Faculty of Medicine, between January 2021 and December 2023. Ethical approval was obtained from the Ethics Committee of Uludağ University, protocol number 2024-5/12, and all procedures adhered to the principles of the Declaration of Helsinki. Written informed consent was waived due to the retrospective nature of the study. All patients in our cohort were retrospectively reviewed through institutional electronic records for a minimum of 12 months postoperatively.

We enrolled a total of 52 consecutive patients undergoing anatomical lung resection and radical lymphadenectomy. To ensure procedural consistency and minimize inter-surgeon variability, the same three thoracic surgeons (ASB, EY, HE) were responsible for resections, surgical hemostasis, and chest closures in every case. The exclusion criteria were as follows: non-anatomical lung resections or the absence of systematic lymph node dissection; a history of coagulopathy or bleeding diathesis; preoperative oral antithrombotic therapy not discontinued at least 7 days prior to surgery; severe renal failure (serum creatinine > 2.0 mg/dL); a history of thromboembolic events; reoperations or extensive pleurectomy/decortication; intraoperative requirement for systemic antifibrinolytics or heparinization (e.g., pulmonary artery clamping); postoperative blood transfusions unrelated to surgical blood loss; and extended resections.

### 2.2. Surgical Technique and Group Allocation

All patients underwent anatomical pulmonary resection via video-assisted thoracoscopic surgery (VATS), thoracotomy, or hybrid approaches. Following the completion of lung resection and mediastinal lymph node dissection, meticulous hemostasis and aerostasis were confirmed. No intraoperative hemostatic agents were utilized in any of the patients.

**Figure 1:**
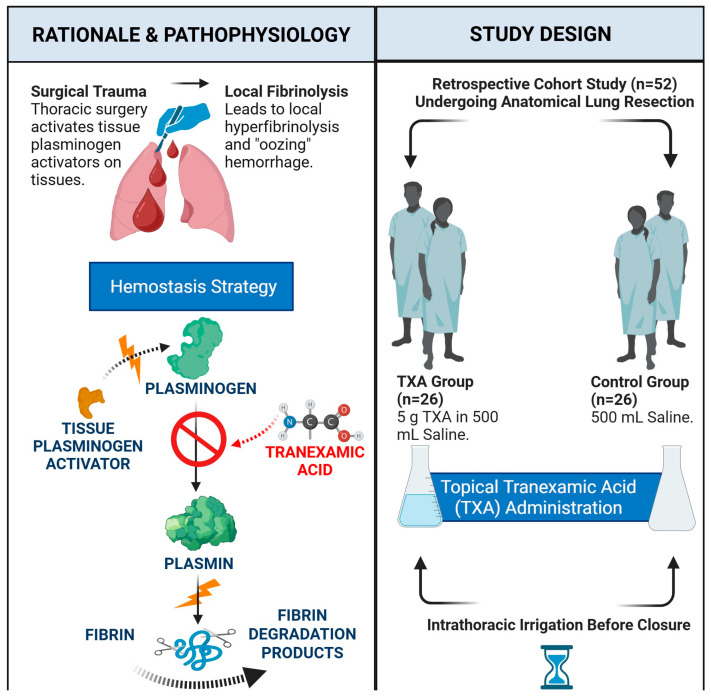
Schematic representation of the study design and the t-TXA application protocol.

The study cohort was divided into two groups based on a temporal shift in our institutional practice protocol. Figure 1 provides a schematic representation of the study design and t-TXA application protocol used.

- Control Group (n = 26): Between January 2021 and June 2022, our routine surgical practice for patients undergoing lung resection exclusively utilized a 500 mL normal saline lavage to check for air leaks and hemostasis before closure.
- t-TXA Group (n = 26): This group consisted of patients operated on between July 2022 and December 2023. Following a departmental protocol update, a high-volume topical intrathoracic TXA lavage (5 g in 500 mL saline) was standardized for all eligible patients to ensure hemostasis and evaluate air leaks prior to thoracic closure.

This temporal allocation was strictly adhered to, without any selection based on individual surgeon preference, intraoperative findings, or specific clinical criteria. Although these two time-based cohorts were largely balanced in terms of baseline clinical and surgical characteristics, they differed significantly regarding age, which was subsequently addressed in our exploratory analyses. The solution was retained in the pleural cavity for an average of 5 min before the placement of chest tubes. Chest tubes (single 28-Fr) were connected to a standard underwater seal system with a suction pressure of −20 cm H_2_O.

### 2.3. Perioperative Management and ERAS Protocol

A uniform perioperative management regimen, termed the Enhanced Recovery After Surgery (ERAS) protocol adaptation period, was implemented for all study participants to ensure consistency in postoperative recovery. All patients received multimodal analgesia, including intraoperative intercostal nerve blocks and postoperative paracetamol/nonsteroidal anti-inflammatory drug combinations, to minimize opioid consumption. A standardized mobilization protocol was followed, with patients encouraged to sit out of bed on the day of surgery and achieve early ambulation by POD 1. Chest tubes were managed according to a strict algorithm. Removal criteria were defined as follows: (I) drainage volume < 200 mL/24 h, (II) the absence of any air leak, and (III) full lung re-expansion confirmed via chest X-ray. Chest tube drainage was monitored hourly over the first 6 postoperative hours and thereafter on a daily basis.

Red blood cell (RBC) transfusion was administered when intraoperative or postoperative hemoglobin levels dropped below 8 g/dL and/or hematocrit fell below 25%. Notably, these transfusions were exclusively triggered by acute postoperative hemoglobin declines following surgical blood loss; patients did not receive blood products solely for the correction of pre-existing chronic or chemotherapy-induced anemia. Fresh frozen plasma was transfused if postoperative blood loss reached 150 mL/h for three consecutive hours or if the international normalized ratio (INR) exceeded 1.8. For cases involving persistent blood loss and a platelet count below 80 × 10^9^/L, platelet concentrates were administered. Reoperation for hemostasis was indicated by chest drainage exceeding 300 mL/h for two consecutive hours, 200 mL/h drainage associated with hemodynamic instability, or radiographically confirmed massive pleural effusion. Discharge decisions were based on objective, predefined clinical criteria, independent of study group assignments.

### 2.4. Data Collection and Follow-Up

Institutional records were retrospectively reviewed to analyze a standardized 12-month follow-up period. Data from outpatient assessments—including chest radiography, laboratory evaluations, and quarterly computed tomography scans for malignancy—at months 1, 3, 6, and 12 were extracted from the electronic database. Clinical outcomes, including adverse events, thromboembolic complications, and mortality, were systematically retrieved from the hospital system.

### 2.5. Statistical Analysis

Statistical analyses were performed using SPSS Statistics (version 31.0; IBM Corp., Armonk, NY, USA) and AMOS (version 31.0; IBM Corp., Armonk, NY, USA). Continuous variables were evaluated for normality using skewness and kurtosis coefficients; values within the range of ±1.96 were considered indicative of a normal distribution. Descriptive statistics are presented as mean ± standard deviation or median (minimum–maximum) for continuous data and as frequencies (n) and percentages (%) for categorical data. Between-group comparisons were conducted using the independent-samples *t*-test for normally distributed variables and the Mann–Whitney U test for non-normally distributed data. Categorical variables were analyzed using the Chi-squared test. Longitudinal changes in postoperative drainage and laboratory parameters were assessed via repeated-measures ANOVA or the paired-samples *t*-test, with Bonferroni correction applied for multiple comparisons. Correlations were evaluated using Spearman’s rank correlation coefficient (r) and categorized as low (0.00–0.30), moderate (0.30–0.70), or high (0.70–1.00). Given the exploratory nature of this retrospective study, no a priori sample size calculation was performed. Instead, we report 95% confidence intervals for the primary outcome of length of stay (LOS), indicating that the precision of our estimate is acceptable for hypothesis generation. For all analyses, a two-tailed *p* < 0.05 was considered statistically significant.

A full multivariable linear regression adjusting for all potential confounders (e.g., age, surgical approach, resection extent, preoperative chemotherapy) would require at least 10–15 events per variable to avoid overfitting. With only 52 patients, such a model would be unstable and prone to spurious findings. Therefore, we chose a more conservative, exploratory approach: partial correlation analysis to assess the age-adjusted association between t-TXA and outcomes. Partial correlation estimates the strength of a linear relationship between two variables (e.g., t-TXA use and LOS) while statistically controlling for a third variable (age). This method is appropriate for hypothesis generation in small samples but does not provide causal adjustment for multiple confounders. Readers should interpret these results as exploratory.

## 3. Results

### 3.1. Baseline Patient Characteristics

A total of 52 patients were enrolled in this study, 26 patients each in the control and t-TXA group. As Table 1 shows, the groups were largely balanced with respect to sex, surgical approach, resection type, and preoperative anticoagulant use (all *p* > 0.05). Despite this overall baseline balance, age differed significantly between groups (64.9 ± 8.3 vs. 59.8 ± 9.7 years; *p* = 0.046). Given the limited sample size, a full multivariable regression may be unstable; therefore, we performed partial correlation as an exploratory adjustment.

Regarding primary diagnosis, 14 (54%) patients in the control group and 17 (65%) patients in the t-TXA group underwent surgery for lung malignancy. As detailed in Table 1, in the control group, the procedures consisted of lobectomy (n = 21, 80.8%), segmentectomy (n = 3, 11.5%), and bilobectomy/pneumonectomy (n = 2, 7.7%). Similarly, in the t-TXA group, patients underwent lobectomy (n = 20, 76.9%), segmentectomy (n = 4, 15.4%), and bilobectomy/pneumonectomy (n = 2, 7.7%). The extent of resection was well balanced between the two groups.

### 3.2. Clinical Outcomes and Drainage Dynamics

As shown in Table 1, the length of hospital stay was significantly shorter in the t-TXA group (4.20 ± 1.23 days) than in the control group (5.88 ± 2.23 days; *p* = 0.001). Conversely, no significant differences were observed between the groups in terms of chest tube duration. There was no statistically significant difference in RBC transfusion requirements between the groups (*p* = 0.697), and fresh frozen plasma was not administered to any patient in the entire cohort. No instances of reoperation for bleeding, postoperative hemothorax, or any other clinically significant bleeding events requiring transfusion were recorded in either cohort. No clinically significant early adverse effects were observed in either group during the 12-month follow-up period.

Table 2 details the specific perioperative and postoperative drainage volumes across the study cohorts to support our hypothesis regarding drug safety. No significant differences were observed between the tranexamic acid and control groups regarding perioperative (p^1^ = 0.632) or postoperative drainage volumes on days 1, 2, and 3 (p^1^ > 0.05). Although POD 1 drainage was lower in the t-TXA group (189.23 ± 235.06 mL) than in the control group (284.23 ± 169.40 mL), this difference did not reach statistical significance (*p* = 0.101). However, both groups exhibited a significant temporal decline in drainage (p^2a^ < 0.001, repeated-measures ANOVA). Post hoc lettering analysis using the Bonferroni test indicated that perioperative and postoperative day (POD) 1 drainage levels (denoted by a) were significantly different from those on POD 2 and 3 (denoted by b).

### 3.3. Hematological Parameters

**Figure 2:**
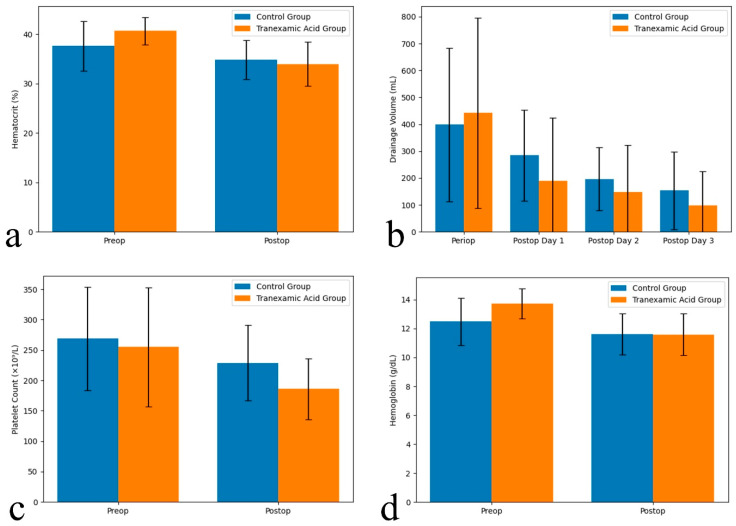
Comparison of parameters between the study groups. (**a**) Hematocrit levels (%), (**b**) postoperative drainage volumes (mL) from the perioperative period to postoperative day 3, (**c**) platelet counts (×10^9^/L), and (**d**) hemoglobin levels (g/dL). Data are presented as mean ± standard deviation.

Figure 2 presents a comparative analysis of hematological parameters and drainage volumes between the t-TXA and control groups, highlighting the trends in hematocrit, hemoglobin levels, platelet counts, and drainage dynamics. Table 2 gives the laboratory parameters, showing that both groups exhibited a significant postoperative decline in hemoglobin, hematocrit, and platelet counts compared to preoperative values (*p* < 0.01). Postoperative platelet counts were significantly lower in the t-TXA group (186.0 ± 50.3 ×10^9^/L) than in the control group (228.8 ± 62.3 ×10^9^/L; *p* = 0.009). No significant changes were observed in INR, aPTT, or fibrinogen levels either between groups or over time.

Patients receiving preoperative chemotherapy exhibited significantly lower hemoglobin and hematocrit levels during both the preoperative (12.43 ± 1.36 g/dL and 37.11% ± 4.17; <0.001) and postoperative (11.09 ± 1.28 g/dL and 32.74% ± 3.72; *p* ≤ 0.003) periods compared to the non-chemotherapy group. Conversely, no significant differences were observed in chemotherapy-induced platelet variations between the groups (preoperative *p* = 0.423; postoperative *p* = 0.944).

### 3.4. Exploratory Correlation Analysis and Long-Term Safety

**Figure 3:**
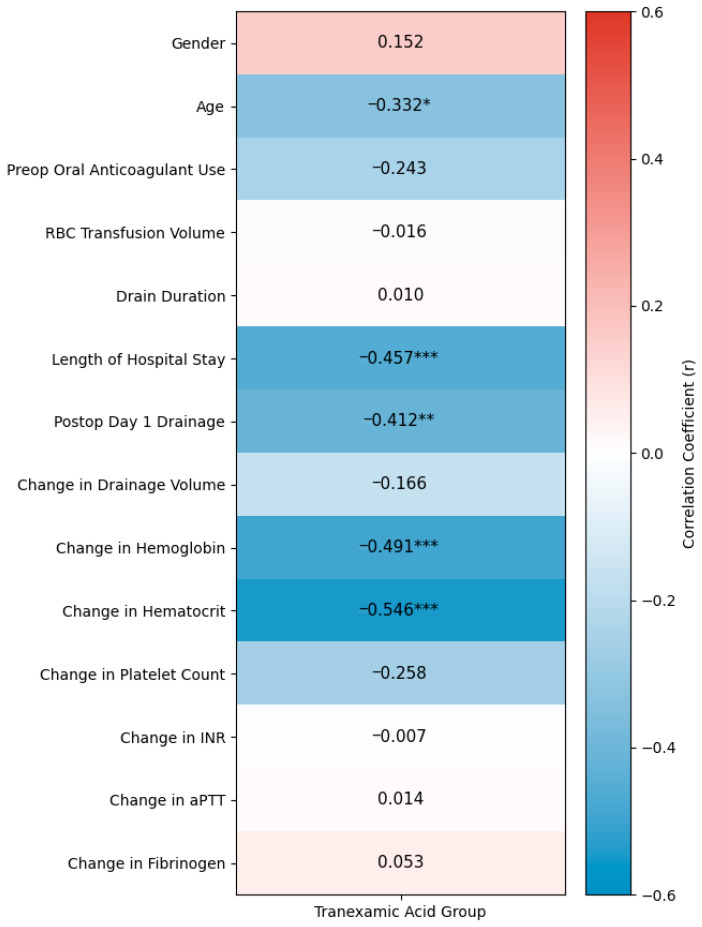
Spearman correlation matrix (vertical orientation) illustrating the exploratory associations between t-TXA administration and selected clinical and laboratory variables. Cells display Spearman correlation coefficients (r) and corresponding statistical significance levels (* *p* < 0.05; ** *p* < 0.01; *** *p* < 0.001). Dark blue shades indicate negative correlations, while dark red shades denote positive correlations; the color intensity reflects the strength (magnitude) of the relationship between variables. These correlation coefficients represent hypothesis-generating associations and should not be interpreted as causal relationships. Abbreviations: RBC, red blood cell; INR, international normalized ratio; aPTT, activated partial thromboplastin time.

Exploratory analysis revealed that t-TXA use was moderately and negatively correlated with both POD 1 drainage volume (r = −0.412; *p* = 0.002) and hospital length of stay (r = −0.457; *p* < 0.001). As shown in Figure 3, the correlation between t-TXA and LOS highlights this potential association. Because age differed significantly between groups, we then performed partial correlation as an adjustment for this variable. After this, the association between t-TXA and reduced LOS remained statistically significant (r = −0.38, *p* = 0.008). However, it is important to explicitly state that these hypothesis-generating results do not compensate for the non-significant primary outcome regarding postoperative drainage (POD 1 drainage, *p* = 0.101). We emphasize that partial correlation only adjusts for age, not for other potential confounders (e.g., surgical complexity, perioperative care changes over time). Therefore, these exploratory findings should be interpreted cautiously and require confirmation in adequately powered prospective trials.

**Figure 4:**
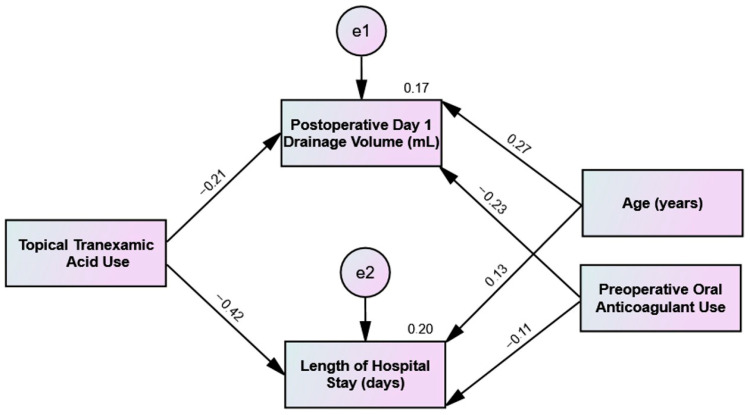
Exploratory path analysis model depicting regression coefficients (β) between t-TXA use, age, preoperative anticoagulant status, and postoperative outcomes. e1 and e2 represent the error terms reflecting the variance in drainage volume and length of stay, respectively, not accounted for by the predictors in the model (e.g., unmeasured confounders). These path coefficients should be interpreted as exploratory trends rather than definitive causal links, given the non-significant primary outcome.

As presented in Figure 4, exploratory path analysis provides a visual representation of relationships between t-TXA administration, patient-specific variables, and clinical outcomes. However, given the cohort size, these path coefficients should be interpreted as exploratory trends rather than definitive causal links. As illustrated, the model indicates a negative exploratory association between t-TXA use and the length of hospital stay (β = −0.42, *p* < 0.001) rather than a definitive causal effect.

All 52 patients completed the 12-month follow-up without attrition. During this period, no thromboembolic events, reoperations for delayed bleeding, or intervention-related late complications were observed in either group. Consequently, one-year overall survival was 100%, with no intervention-related morbidity or mortality.

## 4. Discussion

Postoperative drainage management and the prevention of hemorrhagic complications continue to represent significant challenges in thoracic surgery, directly impacting patient recovery and hospital resources [24,25]. Our study demonstrates that the topical application of TXA serves as a promising adjuvant in optimizing these outcomes.

### 4.1. Hemostatic Efficacy and Hematological Impact

A notable finding in our study concerned the stabilization of hemoglobin and hematocrit levels. Despite the control group having higher preoperative baseline values, postoperative levels between the two groups were equivalent. This equivalence suggests that t-TXA effectively limited the perioperative drop in hemoglobin, supporting its role in reducing occult blood loss [26,27].

Interestingly, postoperative platelet counts were significantly lower in the t-TXA group than in the control group. While TXA typically acts by inhibiting plasminogen activation rather than direct platelet consumption, this difference may reflect a localized modulation of the clotting cascade or a varying physiological response to surgical stress under the influence of high-concentration antifibrinolytics [28,29,30]. Furthermore, our cohort included a high proportion of malignancy cases. Given that cancer patients are constantly in a baseline hypercoagulable state, the use of t-TXA may provide hemostatic balance without triggering adverse systemic thrombotic events, a critical safety consideration in oncologic thoracic surgery [31,32]. The observed decrease potentially reflects an enhanced localized hemostatic process, wherein circulating platelets are more effectively recruited and consumed at the surgical site for fibrin clot formation. Rather than indicating a systemic hematologic deficit, the lower count may serve as an objective surrogate marker for the localized efficacy of our high-volume t-TXA protocol in reinforcing the surgical field. Such localized action is consistent with the drug’s primary mechanism of inhibiting fibrinolysis at the site of tissue trauma without inducing systemic hypercoagulability.

### 4.2. Dosage Rationale and Localized Action

Although recent meta-analyses have confirmed the hemostatic benefits of topical tranexamic acid, the optimal dosage remains unstandardized [33]. The methodology utilized in this study—5 g of TXA diluted in 500 mL of saline—differs from the common literature standard established by Dell’Amore et al. [20], who utilized 1 g–5 g in 100–250 mL. We argue that by harnessing a 5 g high-volume regimen to address the vast contact surface area of the pleural cavity, our lavage/irrigation approach offers a distinct mechanical and therapeutic advantage.

A volume of 500 mL was chosen to ensure comprehensive coverage of the extensive pleural surface area, which is significantly larger than the pericardial cavity, where lower volumes (100–250 mL) are typically used. This high-volume approach allows the TXA solution to reach multiple fibrinolytic hotspots that may be missed with smaller-volume applications. By ensuring this extensive contact, the solution effectively blunts hyperfibrinolysis at its source by locally inhibiting tissue-type plasminogen activators (t-PAs) [34,35].

While intravenous TXA is seen as the gold standard in major surgery, its systemic use in thoracic procedures raises concerns regarding neurotoxicity (e.g., seizure risk) and thromboembolic events [33]. Despite this lack of international consensus, the topical route inherently provides a broad safety margin. Topical intra-pleural application achieves a significantly higher local concentration than systemic doses while minimizing plasma absorption. This theoretically eliminates the risk of TXA-induced seizures—caused by gamma-aminobutyric acid type A and glycine receptor inhibition—while exerting maximum antifibrinolytic activity where it is most needed [11].

### 4.3. Clinical Implications for Fast-Track Surgery

Our comparative analyses suggest that the intervention can offer clinical benefits. The application of high-volume t-TXA was associated with a significant reduction in the mean length of hospital stay. Furthermore, significant negative correlations were observed between t-TXA use and both POD 1 drainage and overall hospital stay. Coupled with the observed stabilization of postoperative hemoglobin levels—which indicates a reduction in occult pleuroparenchymal bleeding—our findings suggest a potential association that should be interpreted cautiously. We emphasize that these exploratory analyses are hypothesis-generating only and cannot be treated as a substitute for a statistically significant primary endpoint. The observed correlations, while intriguing, require confirmation in adequately powered prospective trials. Nevertheless, this association points to a hypothesized synergistic recovery pathway, with effective local hemostasis facilitating a sequence of favorable perioperative outcomes, as follows:

1. t-TXA Application: This promotes early fibrin stabilization on the pleuroparenchymal surfaces.
2. Reduced Drainage: Despite a non-significant drainage reduction, the shorter LOS in the t-TXA group suggests a cumulative clinical benefit.
3. Early Decannulation: Our approach facilitates earlier chest tube removal.
4. Accelerated Mobilization: Attenuated pain and earlier mobilization facilitate expedited discharge [36,37].

From a health economics perspective, reducing LOS by even a single day significantly lowers hospital costs and reduces the risk of nosocomial infections [38]. Our correlation analysis further confirms this, showing a moderate negative relationship between t-TXA use and both POD 1 drainage and LOS, suggesting a potential association that warrants prospective clinical investigation.

Currently, the routine use of t-TXA in thoracic surgery remains an emerging practice rather than a standard adjuvant. Our findings suggest that this protocol may serve as a preliminary step toward optimizing postoperative care, as follows:

- Practicality: t-TXA is an inexpensive agent with a high return on investment via LOS reduction.
- Efficacy: It effectively manages the three pillars of drainage volume, transfusion avoidance in high-risk groups, and LOS [5,11,39].
- Safety: No increased risk of infection (empyema) or unwanted adhesion formation was observed, even in patients where reoperation might be considered.

Our retrospective review of 12-month follow-up data confirms that high-volume topical TXA lavage demonstrates long-term safety, with no recorded thromboembolic events or intervention-attributable mortality. By utilizing the pleural membrane as a natural barrier to limit systemic exposure, this localized hemostatic approach may facilitate fast-track recovery without compromising long-term clinical outcomes.

### 4.4. Limitations

Several methodological limitations regarding confounding control warrant consideration. Firstly, although most baseline variables were balanced, the significant age difference between groups necessitated the use of partial correlation as an exploratory adjustment. However, this approach only accounts for linear effects and may not capture other potential confounders, such as surgical complexity or unmeasured factors such as frailty and intraoperative hemodynamics. Secondly, our modest sample size precluded a comprehensive multivariable regression model; including more than 3–4 covariates would have increased the risk of overfitting and yielded unstable estimates. Thirdly, the temporal allocation of the cohorts—with the control group preceding the t-TXA group—may introduce period effects, such as evolving ERAS protocols and analgesic refinements, for which we could not fully adjust. Consequently, these associations should be interpreted as hypothesis-generating rather than as definitive causal evidence. Additionally, although TXA is widely recognized as a low-cost intervention, a formal cost-effectiveness analysis was not performed to quantify the economic impact of the high-volume protocol relative to the reduced length of hospital stay [5].

A notable constraint is the lack of pharmacokinetic data. The absence of real-time coagulation monitoring (e.g., thromboelastography or rotational thromboelastometry) and plasma TXA concentration measurements limits our ability to quantitatively assess the extent of systemic absorption or its specific impact on the systemic coagulation profile [40,41]. Finally, the relatively short monitoring window and the small study population may be insufficient to definitively assess long-term safety and the incidence of rare thromboembolic events, particularly in this prothrombotic oncologic cohort [19,20,42].

## 5. Conclusions

In conclusion, our preliminary findings suggest that high-volume topical tranexamic acid lavage is associated with a trend toward reduced postoperative drainage and a significantly shortened hospital stay following major lung resection. Furthermore, our 12-month follow-up confirmed a favorable safety profile with no adverse events or mortality. While these results point toward a potential synergistic benefit in terms of enhanced recovery protocols, the retrospective nature and modest sample size of this study preclude us from definitively recommending clinical standardization or routine use.

These observations should be interpreted as preliminary and hypothesis-generating. This underscores the need for well-powered, multi-center randomized controlled trials to establish the long-term safety and practical utility of this intervention in thoracic oncology. Nevertheless, we can deduce that topical tranexamic acid represents a potential adjuvant strategy for optimizing perioperative outcomes in fast-track surgical settings.

## Figures and Tables

**Table 1:** Comparison of patient demographic, surgical, and clinical characteristics according to topical tranexamic acid application. Categorical variables are presented as n (%), and continuous variables are presented as mean ± standard deviation (SD). *p*-values indicate between-group comparisons. Abbreviations: RBC, red blood cell; t-TXA, topical tranexamic acid; VATS, video-assisted thoracoscopic surgery. * *p* < 0.05, ** *p* < 0.01.

Variable	Control (n = 26)	t-TXA (n = 26)	*p*-Value
Sex, n (%)			0.463
Male	23 (88.5)	20 (76.9)	
Female	3 (11.5)	6 (23.1)	
Age (years), mean ± SD	64.92 ± 8.33	59.77 ± 9.74	0.046 *
Surgical approach, n (%)			0.312
VATS	12 (46.2)	17 (65.4)	
Open	11 (42.3)	6 (23.1)	
Hybrid	3 (11.5)	3 (11.5)	
Type of resection, n (%)			0.664
Segmentectomy	3 (11.5)	4 (15.4)	
Lobectomy	21 (80.8)	20 (76.9)	
Bilobectomy/pneumonectomy	2 (7.7)	2 (7.7)	
Preoperative treatment, n (%)			
Chemotherapy	16 (61.5)	13 (50.0)	0.577
Radiotherapy	3 (11.5)	4 (15.4)	1.000
Preoperative oral antithrombotic, n (%)	12 (46.2)	6 (23.1)	0.145
Need for additional chest tube, n (%)	1 (3.8)	1 (3.8)	1.000
Postoperative outcomes			
Lymph node stations dissected, mean ± SD	3.81 ± 1.20	3.50 ± 1.30	0.380
RBC transfusion (units), mean ± SD	0.77 ± 1.14	0.65 ± 0.98	0.697
Drainage duration (days), mean ± SD	6.04 ± 5.69	8.38 ± 7.37	0.205
Length of hospital stay (days), mean ± SD	5.88 ± 2.23	4.20 ± 1.23	0.001 **
Postoperative complications, n (%)	13 (50.0)	13 (50.0)	1.000
90-day mortality, n (%)	1 (3.8)	1 (3.8)	1.000

**Table 2:** Comparison of perioperative drainage and pre- and postoperative laboratory parameters between groups. Data are presented as mean ± SD and median (minimum–maximum). Statistical significance was defined as *p* < 0.05 and *p* < 0.01. p^1^ indicates intergroup comparisons at each time point (independent-samples *t*-test). p^2a^ represents changes over time within each group (repeated-measures ANOVA with Bonferroni correction). p^2b^ denotes within-group comparisons between specific time points (paired-samples *t*-test). a and b are superscript letters that denote specific comparisons. Abbreviations: aPTT, activated partial thromboplastin time; INR, international normalized ratio; SD, standard deviation; t-TXA, topical tranexamic acid.

Variable	Time Point	Control (n = 26) Mean ± SD; Median (Min–Max)	t-TXA (n = 26) Mean ± SD; Median (Min–Max)	p^1^-Value
Drainage (mL)	Perioperative	398.85 ± 285.84; 350 (30–1100) ^a^	441.92 ± 354.66; 325 (10–1300) ^a^	0.632
	Postop day 1	284.23 ± 169.40; 250 (50–700) ^a^	189.23 ± 235.06; 100 (50–1050) ^a^	0.101
	Postop day 2	196.54 ± 117.10; 200 (0–450) ^b^	148.85 ± 172.96; 125 (0–600) ^b^	0.250
	Postop day 3	153.85 ± 144.17; 10 (0–600) ^b^	99.04 ± 125.79; 0 (0–400) ^b^	0.150
	p^2a^	<0.001	<0.001	
Hemoglobin (g/dL)	Preoperative	12.49 ± 1.63; 12.45 (9.5–15.4)	13.73 ± 1.05; 13.7 (11.5–15.8)	0.002
	Postoperative	11.60 ± 1.42; 11.55 (8.5–14.3)	11.59 ± 1.43; 11.55 (8.8–16.0)	0.969
	p^2b^	<0.001	<0.001	
Hematocrit (%)	Preoperative	37.59 ± 4.99; 37.55 (27.3–45.6)	40.64 ± 2.80; 40.7 (32.8–45.6)	0.009
	Postoperative	34.82 ± 3.97; 35.05 (26.4–42.8)	33.96 ± 4.44; 33.95 (25.4–48.1)	0.466
	p^2b^	<0.001	<0.001	
Platelet count (×10^9^/L)	Preoperative	268.7 ± 85.52; 256.4 (138.0–480.4)	254.9 ± 98.32; 228.7 (115.5–594.0)	0.592
	Postoperative	228.8 ± 62.32; 234.6 (122.8–391.0)	186.0 ± 50.31; 184.2 (98.6–356.0)	0.009
	p^2b^	0.005	<0.001	
INR	Preoperative	0.95 ± 0.06; 0.94 (0.84–1.05)	0.93 ± 0.06; 0.92 (0.83–1.10)	0.234
	Postoperative	0.99 ± 0.08; 1.00 (0.88–1.20)	0.95 ± 0.09; 0.90 (0.80–1.10)	0.171
	p^2b^	0.101	0.253	
aPTT (s)	Preoperative	24.20 ± 2.31; 24.15 (19.9–28.4)	24.30 ± 1.93; 24.4 (19.2–26.9)	0.874
	Postoperative	28.67 ± 6.55; 26.0 (21.9–39.6)	25.69 ± 3.85; 25.8 (18.5–33.4)	0.092
	p^2b^	0.108	0.053	
Fibrinogen (mg/dL)	Preoperative	397.0 ± 109.25; 364 (282–613)	336.18 ± 85.91; 325 (222–547)	0.144
	Postoperative	381.12 ± 133.99; 347 (163–771)	371.35 ± 121.11; 361 (157–584)	0.817
	p^2b^	0.521	0.906	

## Data Availability

Data are our property and are available among members of our institution.
